# Lifetime Occupational Physical Activity and Musculoskeletal Aging in Middle-Aged Men and Women in Denmark: Retrospective Cohort Study Protocol and Methods

**DOI:** 10.2196/resprot.2191

**Published:** 2012-07-27

**Authors:** Anne Møller, Ole Steen Mortensen, Susanne Reventlow, Peder Georg Skov, Johan Hviid Andersen, Tine Steen Rubak, Åse Marie Hansen, Lars L Andersen, Rikke Lund, Merete Osler, Ulla Christensen, Kirsten Avlund

**Affiliations:** 1 Department of Occupational MedicineKøge HospitalKøgeDenmark; 2 The Research Unit for General Practice and Section of General PracticeDepartment of Public HealthUniversity of CopenhagenCopenhagenDenmark; 3 National Research Centre for the Working EnvironmentCopenhagenDenmark; 4 Department of Occupational and Environmental MedicineBispebjerg HospitalCopenhagenDenmark; 5 Danish Ramazzini CenterDepartment of Occupational MedicineRegion HospitalHerningDenmark; 6 Section of Social MedicineDepartment of Public HealthUniversity of CopenhagenCopenhagenDenmark; 7 Danish Aging Research CenterUniversities of Southern Denmark, Aarhus and CopenhagenCopenhagenDenmark; 8 Research Centre for Prevention and HealthGlostrup University HospitalGlostrupDenmark; 9 Center for Healthy AgingUniversity of CopenhagenCopenhagenDenmark

**Keywords:** Occupational exposure, work load, physical fitness, musculoskeletal system, aging

## Abstract

**Background:**

Physical function is essential for performing most aspects of daily life and musculoskeletal aging leads to a decline in physical function. The onset and rate of this process vary and are influenced by environmental, genetic, and hormonal factors. Although everyone eventually experiences musculoskeletal aging, it is beneficial to study the factors that influence the aging process in order to prevent disability. The role of occupational physical activity in the musculoskeletal aging process is unclear. In the past, hard physical work was thought to strengthen the worker, but current studies in this field fail to find a training effect in jobs with a high level of occupational physical activity.

**Objective:**

The aim of this study is to examine the influence of lifetime occupational physical activity on physical function in midlife. The study follows the “occupational life-course perspective,” emphasizing the importance of occupational exposures accumulated throughout life on the musculoskeletal aging process taking socioeconomic and lifestyle factors into consideration.

**Methods:**

This study is a retrospective cohort study including a cross-sectional measurement of physical function in 5000 middle-aged Danes. Data was obtained from the Copenhagen Aging and Midlife Biobank (CAMB) which is based on three existing Danish cohorts. Using questionnaire information about the five longest-held occupations, the job history was coded from the Danish version of the International Standard Classification of Occupations (D-ISCO 88) and a job exposure matrix containing information about occupational physical activity in Danish jobs was applied to the dataset. The primary outcomes are three tests of physical function: handgrip strength, balance, and chair rise. In the analyses, we will compare physical function in midlife according to accumulated exposure to high levels of occupational physical activity.

**Conclusions:**

We have a unique opportunity to study the influence of work on early musculoskeletal aging taking other factors into account. In this study, the “healthy worker effect” is reduced due to inclusion of people from the working population and people who are already retired or have been excluded from the labor market. However, low participation in the physical tests can lead to selection bias.

## Introduction

Physical function is essential for performing most aspects of daily life and it is a predictor of morbidity and mortality [1,2]. Musculoskeletal aging leads to a decline in physical function [3], the onset and rate of which vary and are influenced by environmental, genetic, and hormonal factors [4]. Leisure-time physical activity is important for maintaining physical function and is recommended by authorities in many countries [5], but the role of occupational physical activity (OPA) is more controversial [6]. Until the 1980s, manual workers were considered stronger than non-manual workers because of OPA. Since then, muscle strength and endurance have been shown to be lower in manual workers than in non-manual workers [7-10]. The absence of an observed training effect of OPA on physical function has been explained by a lack of an optimal combination of intensity, frequency, and duration of job tasks [7,11].

Since the 1980s, few studies have focused on prolonged exposure to strenuous physical work as a predictor of loss of muscle strength and impaired physical function. One study found no association between lifetime OPA and handgrip strength [11], but other studies have shown a training effect of OPA on shoulder muscle strength [12] and physical capacity in the upper extremities [13]. Three studies in older people with a history of manual labor showed that overall lifetime OPA may be associated with significantly higher rates of disability, lower physical function, and reduced muscular strength [14-16]. Two factors influence the associations found in these studies of accumulated physical activity and later physical function: exposure assessment and confounding factors.

The exposure assessment is essential. Most of the studies previously cited rely on self-reports of workload because self-reports provide the simplest and most cost-effective method of measuring physical exposure in large epidemiological studies [17]. Although the validity of self-reports vary [18], they are useful for detecting relative differences in physical workload among occupational groups. To supplement self-reports of physical exposure, expert judgments and job exposure matrices (JEMs) have been used in occupational epidemiology [19,20]. JEMs are databases based on expert judgments, registers, or measurements and use coded job titles to assign exposures in epidemiologic studies [21]. They are useful for retrospective exposure assessment in population-based studies in which many types of jobs are represented. Several research groups have used expert ratings and established JEMs for assessment of physical exposure [22,23], but the imprecise definition of OPA and the lack of accurate measurements can affect the validity. The use of a panel of experts for assessment of exposure improves the validity of the judgments [24], but misclassification is still possible because a JEM is a group-based assessment and individual differences in exposures because of variation in job tasks among people with the same job title and differences in ergonomics and capacity are not taken into account [25]. A recent study found high validity of reported job histories comparing information from questionnaires and interviews, whereas self-reports of work-life OPA levels showed varying validity (Møller et al, unpublished data, 2012). Therefore, this study uses a JEM on occupational physical activity based on expert ratings to supplement the exposure assessment.

In the previously mentioned studies, differences in physical function among older people could be attributed to confounding factors throughout life. Using a life-course perspective on aging and functional decline, factors such as socioeconomics, lifestyle, and genetics are relevant to take into consideration [4]. However, it is not always possible to follow trajectories of confounding factors in life-course analyses although they can influence outcomes such as physical function in midlife [26-28].

There has been little focus on occupational exposures in life-course studies of physical function [29]. Thus, it is not known how occupational exposures during the course of life influence the musculoskeletal aging process and the decline in physical function. Our hypothesis is that a high level of OPA affects the timing and/or the rate of the musculoskeletal aging process. In this study, the term “occupational life-course perspective” is used and the aim is to examine the influence of lifetime occupational physical activity on physical function in midlife.

## Methods

### Study Design

This study is a retrospective cohort study including a cross-sectional measurement of physical function in midlife. Data will be obtained from the Copenhagen Aging and Midlife Biobank (CAMB) [30], which is based on three existing Danish cohorts aimed at determining the importance of prenatal and perinatal factors, factors in childhood, and factors in early adulthood for early signs of aging in late midlife. Physical examinations of the cohort are planned for the future, but not yet funded.

### Study Population

This study utilizes data from two of the three CAMB cohorts: The Metropolit Cohort and the Danish Longitudinal Study on Work, Unemployment and Health. From these cohorts, 12,656 middle-aged men and women living in Denmark were invited to participate. Data collection took place between April 2009 and March 2011. Of the initial 12,656 invitations, 39.97% (5059/12,656) answered the postal questionnaire and 30.48% (3858/12,656) attended the examination. Presently, the CAMB database is being prepared for analysis. The analyses will begin in the spring of 2012 when the database will be available for further research and the job exposure matrix has been established.

### Description of the Cohorts

The Metropolit Cohort is defined as the 11,532 men born in 1953 in the Copenhagen Metropolitan area and living in Denmark in 1968. The cohort has been described in detail elsewhere [31]. Data from birth certificates, including information on dimensions at birth and the father’s occupational status at the time of birth, were manually collected for all members of the original study population in 1965. That same year, 7987 (69.26%) of these males participated in a school-based survey that included a questionnaire administered by their class teachers. The questionnaire included tests of cognition and questions regarding leisure-time activities and social aspirations. In addition, data from conscription board examinations between 1971-1976, including measurements of height, weight, and cognitive function, were collected from archives in 2004. In 2004, 6292 members of the cohort responded to a health questionnaire. The Metropolit Cohort provides a unique opportunity to study early biological and social influences on the development of a number of social and health outcomes [32-34].

The Danish Longitudinal Study on Work, Unemployment and Health is a prospective population study that began with a baseline postal survey in the spring of 2000 in a stratified random sample (n = 15,227) consisting of two population groups: (1) individuals between 40 and 50 years by October 1, 1999 (7588/11,082, response rate 68.47%), and (2) individuals between 36 and 54 years who were unemployed at least 70% of the time between October 1, 1996 and October 1, 1999 (2350/4200, response rate 55.95%). Both samples were drawn initially from the “Anvendt Kommunal Forskning” (AKF) Longitudinal Register maintained by the Danish Institute of Governmental Research. The AKF Longitudinal Register comprises 100% of the Danish population aged 15 years and older. Data on non-participation was derived from the register. Non-participants included a significantly higher proportion of men, non-native-born Danes, individuals living on transfer income, and individuals with lower education levels (untrained or semi-skilled). In 2006, a follow-up questionnaire was sent to the surviving respondents, now aged 44-62 years (n = 8916) and a completed questionnaire was returned by 6151 respondents (response rate 68.99%). Data included a number of demographic, socioeconomic, psychosocial, and behavioral measures because socioeconomics and the consequences of unemployment were two of the main fields of investigation [35-37].

### Conceptual and Analytical Model

**Figure 1:**
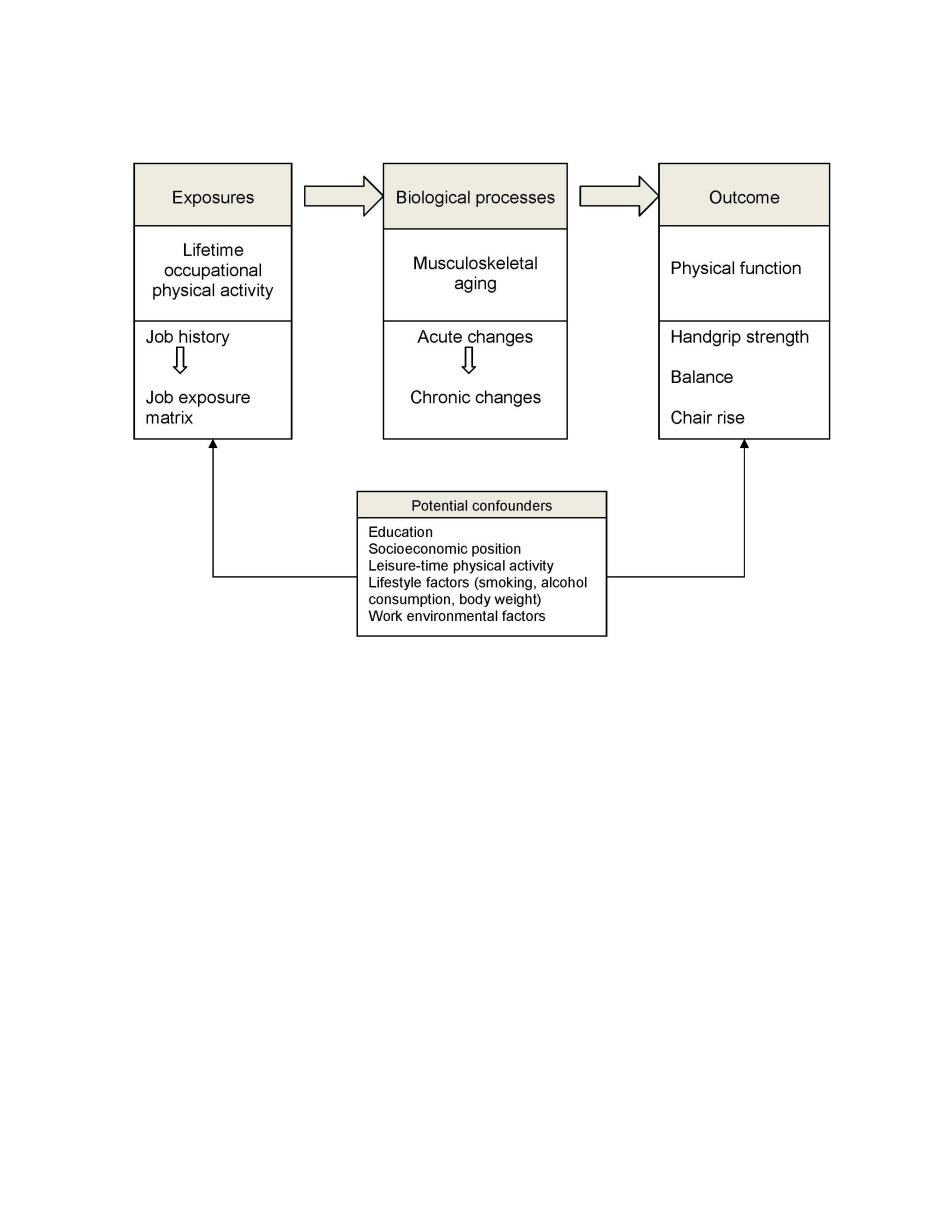
Conceptual model of study.

Figure 1 illustrates the conceptual model used in this study. The research hypothesis is that prolonged exposure to high levels of OPA is associated with lower levels of physical function in midlife. The physiological link between exposure and outcome is the underlying biological processes, where acute changes in the musculoskeletal system turn chronic because of insufficient time for recovery [38,39] and a cumulative effect of physical wear and tear over the years influences the onset and rate of the musculoskeletal aging process [13]. At the same time, other factors throughout the course of life are associated with the exposure to occupational physical activity and physical function in midlife, which are potential confounders in our conceptual model.

### Exposure Assessment

Occupational physical activity is the main exposure in this study. We define occupational physical activity as “work including mostly standing and walking at work combined with daily lifting of heavy burdens.”

#### Self-reported Measures

The questionnaire provides data on the five longest occupations held plus the current occupation. The job titles were coded using the 1988 revision of the Danish version of the International Standard Classification of Occupations (D-ISCO 88) registration system by a coder with a broad knowledge of the Danish labor market. The International Standard Classification of Occupations (ISCO) was developed by the International Labour Office in 1958 and is a standardized classification and rating system of job types according to skills and education requirements [40].The D-ISCO contains classifications for more than 2000 Danish job titles as four-digit codes and it is used primarily for statistical analysis and research.

The questionnaire provides information about exposure during working life to dust, noise, chemicals, heavy lifting, working with the back bent, about the psychosocial work environment, and OPA. The OPA is categorized into four groups: sedentary work (eg, office work); mainly standing and walking at work (eg, teachers or machine operators); moderate physical exertion (eg, car mechanics or cooks); and hard physical work including lifting and pushing/pulling (eg, furniture movers or bricklayers). For all types of exposures, the respondent has to include a summation of their years of exposure.

#### Job Exposure Matrix

A job exposure matrix, the occupational physical activity matrix (OPA matrix), was applied to the dataset. The OPA matrix is based on an existing Danish job exposure matrix called the Knee-Hip Matrix [41] that is based on expert judgment of physical exposures associated with risk of osteoarthritis (eg, sitting, standing/walking, whole-body vibration, kneeling, and lifting of heavy objects). Firstly, all jobs in the D-ISCO classification considered more than minimally exposed to at least one of the exposures of interest were collapsed into homogeneous exposure groups (HEGs). Of the 2227 possible, 689 job titles were collapsed into 121 HEGs containing from 1-34 different occupational titles. For example, the HEG “people working in the printing industry” includes bookbinders, machine operators, and printers; “people working with food preparation in different kitchens” includes different types of cooks and managers in cafés and cafeterias.

#### Expert Rating

In keeping with international recommendations for expert ratings, a panel of five raters (experienced occupational physicians) independently assessed the 122 HEGs. They rated the duration of sitting, standing/walking, kneeling, and whole-body vibration throughout a normal working day. Furthermore, a rating of daily lifting (kg/day) and number of lifts over 20 kg per day were assigned to each HEG. A final consensus meeting was held to discuss outliers and discrepancies in the ratings and the method was validated internally and externally [41].

#### The OPA Matrix

The division of job titles in the HEGs and the average rating of the experts on physical activity (eg, hours standing/walking per day and lifting frequency and intensity per day) came from the Knee–Hip Matrix. Job groups not included in the HEGs were assigned as “unexposed.” Years of exposure to standing and walking and lifting were calculated according to the following definitions: (1) standing year (SY) defined as 6 hours of standing and walking at work each day in 1 year; (2) lifting year (LY) defined as lifting more than 20 kg at least 10 times per day in 1 year; and (3) ton year (TY) defined as 1000 kg of heavy lifting per day in 1 year. Each participant’s job history was converted to D-ISCO job titles covering the 0–5 previous longest occupations. Finally, data on exposure from the OPA matrix was assigned to the respective job titles and a summation of exposure was calculated.

### Outcome Assessment

#### Test Protocol

Participants in CAMB attended an examination at the National Research Centre for the Working Environment (NRCWE), which involved a review of the previously completed questionnaire, measurement of weight and height, a battery of physical tests, cognitive tests, blood sampling, and information about health status with respect to some of the results of the examination. The battery of physical tests included tests of handgrip strength, trunk extension and flexion, jump height, flexibility, chair rise, and balance. General exclusion criteria for participation in the physical tests were high blood pressure, self-reported signs of angina pectoris, and use of prescribed heart/lung medication.

#### Objective Measures of Physical Function

Three signs of early musculoskeletal aging were used as outcome measures: handgrip strength, balance, and chair rise.

Handgrip strength was measured during a maximal voluntary isometric contraction with an electronic version of the Jamar dynamometer [42]. The participant sat upright on a chair with the elbow flexed 90 degrees and was instructed to squeeze the dynamometer as fast and as forcefully as possible. From a total of 3-5 attempts, the highest force value was used as the handgrip strength.

Balance was measured on an Advanced Mechanical Technology, Inc (AMTI) force platform during a one-legged stance with eyes open and arms across the chest [43,44]. The participant focused on a dot on the wall and stood as steady as possible for 30 seconds. Balance was defined as the sway area (95% confidence ellipse measured in cm^2^). A lower sway area indicates better balance. Three 30-second attempts were given and the lowest sway area of the three attempts was used. Some participants were unable to maintain their balance for 30 seconds; therefore, the outcome was also dichotomized as a “yes/no” answer regarding completion of the test.

The ability to rise from a chair was determined by a chair rise test. Participants were instructed to rise and sit as many times as possible over 30 seconds [45]. Only one attempt was given because of the tiring nature of the test. An electronic switch placed under the seat of the chair counted the total number of chair rises.

### Confounders and Intermediate Variables

Information about various confounders was available from the CAMB questionnaire:

1. Chronic diseases. Number of chronic diseases are registered and grouped into three groups: no chronic disease, one chronic disease, and two or more chronic diseases. Relevant diseases were asthma, diabetes, hypertension, angina pectoris, stroke, myocardial infarction, bronchitis, emphysema, osteoarthritis, cancer, anxiety, depression, other psychiatric diseases, and back pain.

2. Pain. A general pain score was calculated from answers about pain levels in 9 parts of the body.

3. Leisure-time physical activity. Information about weekly physical activity during leisure-time was reported in two ways. Duration of housing and gardening work plus walking and bicycling (including transportation to work) is summated and categorized as less than 3 hours per week, 3–6 hours per week, and more than 7 hours a week. Another question included a more specific description of the intensity of physical activity during leisure time and was categorized as low, medium, or high intensity.

4. Smoking. Smoking was reported as smoker/non-smoker including a smoking history in pack years (defined as 20 cigarettes or an equal amount of tobacco smoked each day for 1 year).

5. Alcohol consumption. Alcohol consumption was categorized in units of alcohol per week.

6. Education. School education was categorized into three groups: no exam, primary education, and secondary/higher education. Vocational education was categorized into five groups: unskilled, skilled manual worker, and short, medium, or long further education.

7. Occupational social class. Information about current occupation and education was used to categorize participants into eight socioeconomic classes.

8. Psychosocial work environment. Information about psychosocial work environmental factors (eg, demands, feedback, support, and influence) was also included in the analyses as confounders.

9. Physical measures. Height, weight, and lean body mass were measured at the examination. Body mass index (BMI) was categorized into four groups: <18.5, 18.5–25, 25–30, and >30 kg/m^2^.

### Statistical Analysis

The primary outcome is signs of early musculoskeletal aging, measured as performance in the three physical tests.

The following null-hypothesis will be tested: in 3 tests of physical function, there is no difference between middle-aged Danes according to their level of lifetime occupational physical activity.

Because prior studies hypothesized a positive association between manual workers and handgrip strength, we will analyze handgrip strength in a separate analysis. Analyses will be stratified for gender due to differences in physical capacity.

First univariate analyses of the cumulative exposures to standing and lifting and the associations with the three outcome measures will be calculated using logistic regression analyses. Analyses will be repeated using self-reports of exposure. Afterwards multiple regression analyses with stepwise forward selection of variables will be used.

Dropout analyses will be done with the CAMB database to study attrition by using information on socioeconomic status, health, and lifestyle factors from previous questionnaires and registers.

### Power Calculation

The power calculation is based primarily on the studies of Kuh et al of a British birth cohort of comparable age and size (2797 individuals age 53 years) [29,46]. Work is included as a dichotomized covariate (manual/non-manual) in their multivariate regression analyses. We expect to find larger differences in our study using a more specific exposure assessment. The following power calculations were performed in SAS version 9.2 PROC POWER. It is assumed that 20% of the population has a job history that includes a moderate to high level of OPA, and we are aiming for a power of 90% (beta = .1) with a significance level of 5% (alpha = .05) in the following calculations.

#### Handgrip Strength

Kuh et al found a non-significant difference of 0.3 kg in handgrip strength between manual and non-manual male workers [29]. A significant difference of 4 kg between manual and non-manual workers was found in the Il SIRENTE study [14], in which hard physical work was a primary exposure but included older participants. Presuming a relevant difference of 4 kg and the previous assumptions, 2375 participants are needed to show a statistical difference.

#### Chair Rise Test

Kuh et al measured time to complete 10 chair rises and found a difference of 0.3 sec^-1 ^(SD 3.3) between manual and non-manual workers. This is a small difference. A slightly larger difference of 0.5 sec^-1 ^is more appropriate, so that manual and non-manual workers use 22 and 20 seconds, respectively. Given this and the other assumptions described previously, n is calculated to be 2870.

#### Balance

In the British birth cohorts, balance was tested at home and the results are not comparable to our test of balance using the AMTI platform.

From the power calculations in SAS PROC POWER, we will find significant differences (alpha = .05) between manual and non-manual workers in the three physical tests if we include at least 3000 persons.

## Discussion

Prevention of decline in physical function due to working conditions is important to the individual worker and to society as a whole in order to maintain the ability to work and prevent disability later in life [47]. More knowledge is needed about the associations between lifetime workload and midlife physical function.

Measurements of physical function are more valid measures than self-reports of pain or disability. The three physical tests were chosen to study functional limitations instead of specific diseases. These outcome measures have been used mainly in gerontological studies [1,2]. There is an association between handgrip strength and mortality in elderly people and handgrip strength and mortality due to all causes in midlife have recently been shown to be associated [2].

### Strengths and Limitations

CAMB is a population-based cohort and inclusion is not based on symptoms or diseases [24] which reduces the “healthy worker effect.” Cohort members are invited and included regardless of their status in the labor market. Therefore, the cohort includes middle-aged Danes who are still working, on disability pension, unemployed, sick-listed, or have retired early. All have their occupational history recorded through the questionnaire. However, health and physical capacity during youth is not taken into account in our analyses. We hope to be able to include data on chronic diseases in youth in later analyses in order to study selection into jobs or into the labor market. A low participation in CAMB among those invited can lead to selection bias; therefore, dropout analyses are crucial.

We introduce an individual summation of exposure to occupational physical activity in working life using self-reports of job history and expert judgments of exposure to OPA and the exposure assessment is strengthened by a combination of dose (level of physical activity) and time (duration of occupation) [48]. However, there is a risk of misclassification due to generalization of physical demands in job groups (HEGs) in the JEM. By using the five longest-held jobs plus the present job in the exposure assessment, we take account of the fact that deterioration of physical function may be a chronic process and symptomatic workers are more likely to change jobs from high exposure to low exposure [22,49].

Changes in lifestyle factors and socioeconomics throughout life are not taken into account in this study, but historic data about exposures during childhood and adulthood will be included in future analyses. Because the participants did not have a physical examination during their youth, we cannot make conclusions about causal relationships with respect to changes in physical function.

### Impact of Results

In Danish public opinion, OPA is considered to have detrimental effects on health. Work-related exposure is thought to be the primary cause of decline in physical function and ability to work in midlife. We hope to investigate this using the occupational life-course perspective on musculoskeletal aging and physical function. At the same time, it is important to prevent early exit from the labor market due to demographic changes in the Western world. This study will help pinpoint targets for such prevention strategies.

## Abbreviations

AKF: Anvendt Kommunal Forskning (“Danish Institute of Governmental Research”)
AMTI: Advanced Mechanical Technology, Inc.
BMI: body mass index
CAMB: Copenhagen Aging and Midlife Biobank
D-ISCO: the Danish version of ISCO
HEG: homogeneous exposure groups
ISCO: International Standard Classification of Occupations
JEM: job exposure matrix
LY: lifting year (lifting more than 20 kg at least 10 times per day in a year)
NRCWE: National Research Centre for the Working Environment
OPA: occupational physical activity
SY: standing year (6 hours of standing and walking at work per day in a year)
TY: ton year (1000 kg of heavy lifting per day in a year)
